# Genetic Diversity Analysis of Highly Incomplete SNP Genotype Data with Imputations: An Empirical Assessment

**DOI:** 10.1534/g3.114.010942

**Published:** 2014-03-13

**Authors:** Yong-Bi Fu

**Affiliations:** Plant Gene Resources of Canada, Saskatoon Research Centre, Agriculture and Agri-Food Canada, Saskatoon, SK S7N 0X2, Canada

**Keywords:** Genetic diversity, genotyping-by-sequencing, imputation, missing data, unordered marker data

## Abstract

Genotyping by sequencing (GBS) recently has emerged as a promising genomic approach for assessing genetic diversity on a genome-wide scale. However, concerns are not lacking about the uniquely large unbalance in GBS genotype data. Although some genotype imputation has been proposed to infer missing observations, little is known about the reliability of a genetic diversity analysis of GBS data, with up to 90% of observations missing. Here we performed an empirical assessment of accuracy in genetic diversity analysis of highly incomplete single nucleotide polymorphism genotypes with imputations. Three large single-nucleotide polymorphism genotype data sets for corn, wheat, and rice were acquired, and missing data with up to 90% of missing observations were randomly generated and then imputed for missing genotypes with three map-independent imputation methods. Estimating heterozygosity and inbreeding coefficient from original, missing, and imputed data revealed variable patterns of bias from assessed levels of missingness and genotype imputation, but the estimation biases were smaller for missing data without genotype imputation. The estimates of genetic differentiation were rather robust up to 90% of missing observations but became substantially biased when missing genotypes were imputed. The estimates of topology accuracy for four representative samples of interested groups generally were reduced with increased levels of missing genotypes. Probabilistic principal component analysis based imputation performed better in terms of topology accuracy than those analyses of missing data without genotype imputation. These findings are not only significant for understanding the reliability of the genetic diversity analysis with respect to large missing data and genotype imputation but also are instructive for performing a proper genetic diversity analysis of highly incomplete GBS or other genotype data.

Genotyping by sequencing (GBS) recently has emerged as a promising genomic approach for exploring genetic diversity and association mapping on a genome-wide scale (Huang *et al.* 2009; Elshire *et al.* 2011; Fu and Peterson 2011; Poland and Rife 2012), thanks to the advances in next-generation sequencing technologies (Metzker 2010). Based on the genome reduction with restriction enzymes (Altshuler *et al.* 2000), the GBS approach does not require a reference genome for single nucleotide polymorphism (SNP) discovery, is a one combined step process of marker discovery and genotyping, and provides a rapid, high-throughput, and cost-effective tool for a genome-wide analysis of genetic diversity for a range of nonmodel species and germplasm sets (Poland and Rife 2012; Fu *et al.* 2014). However, one of the unique features associated with GBS is the generation of highly incomplete SNP genotype data (Williams *et al.* 2010; Fu and Peterson 2011, 2012; Poland *et al.* 2012; Fu *et al.* 2014), largely due to low coverage sequencing (Davey *et al.* 2011). The incompleteness could be up to 90% of observations missing (Elshire *et al.* 2011; Fu and Peterson 2011) and is considerably larger than in traditional genetic data with 15% or lower level of missing observations. Such a high incompleteness in genotype data will undoubtedly make genetic analyses difficult and less reliable (Pool *et al.* 2010; Nielsen *et al.* 2011; Crawford and Lazzaro 2012). Thus, it is desirable to assess the accuracy of various population genetic analyses (Marchini and Howie 2010; Rutkoski *et al.* 2013), including those for genetic diversity, with respect to incompleteness in GBS genotype data.

Some genetic analyses of SNP data with low levels of missing observations can provide reliable estimations of genetic parameters, but little is known about the impact when the missing level is up to 90%. Traditionally, when the missing level is 15% or lower, it is common to eliminate genetic markers with incomplete observations, which could result in some information loss (Excoffier and Lischer 2010). To minimize the loss, several imputation methods based on row averages, row medians, and data correlation (Little and Rubin 1987; Horton and Kleinman 2007; Carpenter and Kenward 2013) have been applied to infer missing genotypes for a genetic analysis (Troyanskaya *et al.* 2001; Iwata and Jannink 2010; Weigel *et al.* 2010). The applications based on the haplotype analysis with a reference genome sequence have been quite successful in recovering missing genotypes to empower many genetic analyses such as genome-wide association mapping (Marchini and Howie 2010). However, many GBS data are generated from nonmodel organisms without sequenced genomes, and resulting SNP markers are not ordered along a reference genome or genetic maps, making the haplotype-based imputations not feasible for genetic analyses of GBS data (Poland *et al.* 2012).

Efforts to impute unordered SNP genotype data have been made using regression-based methods such as random forest (RF, Breiman 2001; Stekhoven and Bühlmann 2011) and principal component analysis (PCA)-based tools (Stacklies *et al.* 2007) with the hope of improving genomic selection (Poland *et al.* 2012; Rutkoski *et al.* 2013). These efforts revealed that RF yielded better performance in genomic selection (Rutkoski *et al.* 2013), but it was much slower than PCA-based imputations. However, little attention has been paid to issues associated with unordered missing SNP data and imputation application for a genetic diversity analysis (Williams *et al.* 2010; Fu *et al.* 2014), as current studies on genotype imputations largely considered only the ordered SNP data (Rutkoski *et al.* 2013). Given the increasing use of GBS to explore genetic diversity in nonmodel species (Poland and Rife 2012; Sonah *et al.* 2013; Lu *et al.* 2013) and in 7.4 millions of *ex situ* plant germplasm samples conserved in world genebanks (FAO 2010; Fu and Peterson 2011, 2012), more needs to be learnt about the reliability of a genetic diversity analysis of GBS genotype data (Fu *et al.* 2014).

As allele frequency distributions in GBS data are unknown and may vary for different species, we conducted an empirical assessment of accuracy in genetic diversity analyses of highly incomplete genotype data with imputations. Specifically, this was done with the acquisition of three large SNP genotype data from published corn, rice and wheat databases. Missing data were randomly generated with up to 90% of its observations and then imputed for missing genotypes with three map-independent imputation methods. The genetic diversity analyses of original, missing and imputed data were performed and the corresponding estimates of diversity parameters were compared. It was our hope that this assessment allowed for a better understanding of bias, if any, in the genetic diversity analysis of highly incomplete GBS genotype data. A biased estimation of diversity parameters could mislead the comparison of genetic diversity, invalidate the analysis of genetic structure, or complicate the inference of genetic relationship.

## Materials and Methods

### Original dataset

Since the ideal GBS data set (*i.e.*, a complete GBS genotype data without any missing observations) is currently not available, we acquired three large published genotype data sets of genome-wide SNP markers for this assessment (see supporting information, File S1, File S2, and File S3). The first data set was the original corn genomic data set published by Van Heerwaarden *et al.* (2012), in which 400 diverse corn lines were genotyped with 45,997 polymorphic SNP markers with less than 10% missing data. The second data set was the rice genotype data published by Zhao *et al.* (2010), in which 395 diverse rice lines were genotyped by 1311 polymorphic SNP markers. The third one was a subset of the original genomic data published by Cavanagh *et al.* (2013), in which a worldwide sample of 2994 accessions of hexaploid wheat were genotyped for 90,000 gene-associated SNP markers. For this assessment, we arbitrarily chose the Leaf Rust Panel of 339 diverse wheat lines that were genotyped by 33,526 polymorphic SNP markers. Although genome sequences for corn and rice have been published, this assessment treated the assayed data sets as unordered SNP genotype data.

### Generation of randomly missing data

For each original data set, a simulation was performed to generate 100 sets of randomly missing data with a given missing level by a random selection of observations from the original data set as missing without considering existing missing observations, individual lines and markers. The assessed missing levels ranged from 10 to 90% of the total observations with missing values which were compatible with those reported in GBS genotype data (*e.g.*, Elshire *et al.* 2011; Fu and Peterson 2011, 2012; Fu *et al.* 2014). This was done for all the three original data sets.

### Imputation methods

We assessed three imputation methods in this study, as these imputations have been shown to be the most promising tools to recover missing genotypes (*e.g.*, see Moser *et al.* 2009; Rutkoski *et al.* 2013). The first one is the widely used RF regression procedure (Breiman 2001), and it is based on all available data to predict the missing values for every locus. The RF procedure has been described in detail for imputing missing genotypes for genomic selection (Rutkoski *et al.* 2013) and implemented in the freely available R package “randomForest” (Liaw and Wiener 2002; R Development Core Team 2013). The other two are PCA-based imputation procedures: probabilistic PCA (PP) and nonlinear iterative partial least squares PCA (NI). These two procedures are described in detail and implemented in the freely available R package “pcaMethods” (Stacklies *et al.* 2007). The principle behind these PCA-based imputations is that missing values are initially set to the row averages, and singular value decomposition of the SNP matrix is used to create orthogonal principal components. The principal components, which correspond to the largest eigenvalues, are then used to reconstruct the missing SNP genotypes in the SNP matrix. Nonlinear iterative partial least squares PCA uses the algorithm of nonlinear estimation by iterative partial least squares (Wold 1966) for finding the principal components of the SNP matrix. Note that the PCA-based methods generated estimates on a continuous scale, which can be less than 0 or larger than 2. An ad-hoc binning algorithm was used to assign the PCA-based estimates into distinct genotypes, where homozygous genotype 1 was assigned if the estimate was ≤1.5, the other homozygous genotype 3 if the estimate was >2.5, and the heterozygous genotype 2 when the estimate was >1.5 and ≤2.5. These reasonable assignments were obtained after several verification trials of each crop data set, but they were not optimized as it is required for each PCA-based imputation in each crop data set.

### Genetic diversity analysis

We considered a typical crop genetic diversity analysis with three major diversity components that were aimed to (1) estimate observed and expected heterozygosities (Ho, He) and inbreeding coefficient (F), (2) assess genetic differentiation, and (3) infer genetic relationships among different groups of crop germplasm, respectively. Specifically, estimations of Ho, He, and F were performed following the formulae shown in Box 1.2 of GenAlex Tutorial 1 for co-dominant marker data (Peakall and Smouse 2012). Genetic differentiation among groups of germplasm was estimated with Phi statistics (Φst) following the formulae shown in Box 2.1 of GenAlex Tutorial 2 for co-dominant marker data (Peakall and Smouse 2012). The estimations of genetic diversity and differentiation were done with custom R functions specifically written and tested in the R environment (http://www.r-project.org) with standard genotype data before use. Genetic relationships among different groups of crop germplasm were inferred by obtaining a distance matrix with a custom R function and clustering them based on the neighbor-joining algorithm with the freely available R package “ape” (Paradis *et al.* 2004). The custom R function inputted SNP genotype data for different groups, took into account missing data (if any) to calculate similarity using simple match coefficient (Sokal and Michener 1958), and converted those similarity values into a dissimilarity distance matrix.

### Assessment procedure

The overall assessment procedure for a crop data set started with the input of an original crop data set, generation of its randomly missing data, and imputation for missing genotypes, followed by the genetic diversity analysis of original, missing, and imputed data. For this assessment, nine custom R scripts were specifically written, each involved with each imputation method (RF, PP, NI) and each diversity component (genetic diversity, differentiation, and relationship), and they are available upon request. More specifically, each R script started with (1) an acquisition of an original crop data set; (2) a function of generating a random missing data with a given missing level; (3) a function of a specific diversity analysis; (4) a loop of generating randomly missing data from the whole data set, sampling for a random subset of 130 lines and 1000 markers, imputing missing genotypes of the sampled subset data following specific imputation method as described previously, performing the specific diversity analysis as mentioned previously of the three subset data (original, missing, and imputed), and accumulation of parameter estimates over simulation runs; and (5) ended with related estimation outputs. Note that the loop functioned for each of the nine levels of missing observations (from 10 to 90%) and the number of simulation runs (=100 in this study). The mean and standard deviation of a diversity estimate over 100 runs were obtained, except for the topology accuracy (see below). These nine R scripts were run for each of the crop data sets.

For each simulation run, a random sampling of 130 lines and 1000 markers from the whole randomly missing data set was made, and their corresponding original genotype data were obtained. This step is required to make the assessment practically feasible, as the original crop data sets are large, requiring a large computing capacity and execution time, particularly for RF imputation (see the *Results* section *Imputation accuracy and execution time*). The imputation was made only on the random subset of 130 lines with 1000 markers. Consequently, each simulation run generated a random subset of original, missing (for a given missing level), imputed data for the diversity component analysis. The random subset of 130 lines should reasonably sample the originally defined groups in each crop data set and thus be suitable for the assessment on genetic diversity analysis. In this assessment, we considered the originally defined groups of interest in each crop data set as the known stratifications within each crop. Specifically, 400 corn lines represented four major age groups; 395 rice lines were grouped by Zhao *et al*. (2010) as four of the major subpopulations (*indica*, *aus*, *tropical japonica*, and *temperate japonica*); and 339 wheat lines represented four source groups of wheat leaf rust.

For this assessment, the estimation of heterozygosity and inbreeding coefficient was made above the group level. The functions for estimating both observed and expected heterozygosities took the missing observations into consideration, as the allele frequency estimation was made only from those data without missing genotypes. For the assessment on genetic relationships, each simulation run after the imputation step had an additional random sampling of one individual sample as a representation for each group from the random subset; the corresponding genotype data for the four representative samples were obtained; and the topology of the genetic relationships among the four representative samples was assessed by following the method of Wiens and Tiu (2012). The topology accuracy was determined by counting the number out of 100 missing or imputed data sets with the same neighbor-joining tree of the four representative samples as that from the corresponding original genotype data.

For each diversity estimates, bias was inferred by comparing the estimate from missing or imputed data with that obtained from the original data and calculated, if any, as the percentage of the difference between estimates obtained from missing (or imputed) data and original data over the estimate from the original data.

To assess the impact of sample size on the imputation-based estimation of diversity parameters, we performed extra simulations on the wheat data set with the PP imputation method for four sample size combinations of breeding lines (130, 260) and SNP markers (1000, 10,000). For each sample size combination, each diversity estimate was obtained over 100 simulation runs.

### Imputation accuracy and execution time

The imputation accuracy on imputed genotypes was estimated as the percentage of the imputed genotypes matched with the corresponding original genotypes over all the random missing SNP genotypes for a given missing level in each simulation run. The mean and standard deviation of the accuracy estimate were obtained over 100 simulation runs. This was done for each crop data set, missing level and imputation method. An extra effort was also made to estimate the imputation accuracy on specific imputed genotypes as the percentage of the specific imputed genotypes matched with the corresponding original major (or frequent AA), heterozygous (Aa), or minor (or less frequent aa) genotypes over all the random missing SNP genotypes. During the executions of the custom R scripts in a Linux server (with version of 2.6.18−238.5.1.e15 GNU/Linux), the execution time for each imputation method was also recorded for comparison, as different imputation algorithms may vary in running time.

## Results

### Allele distribution

**Figure 1:**
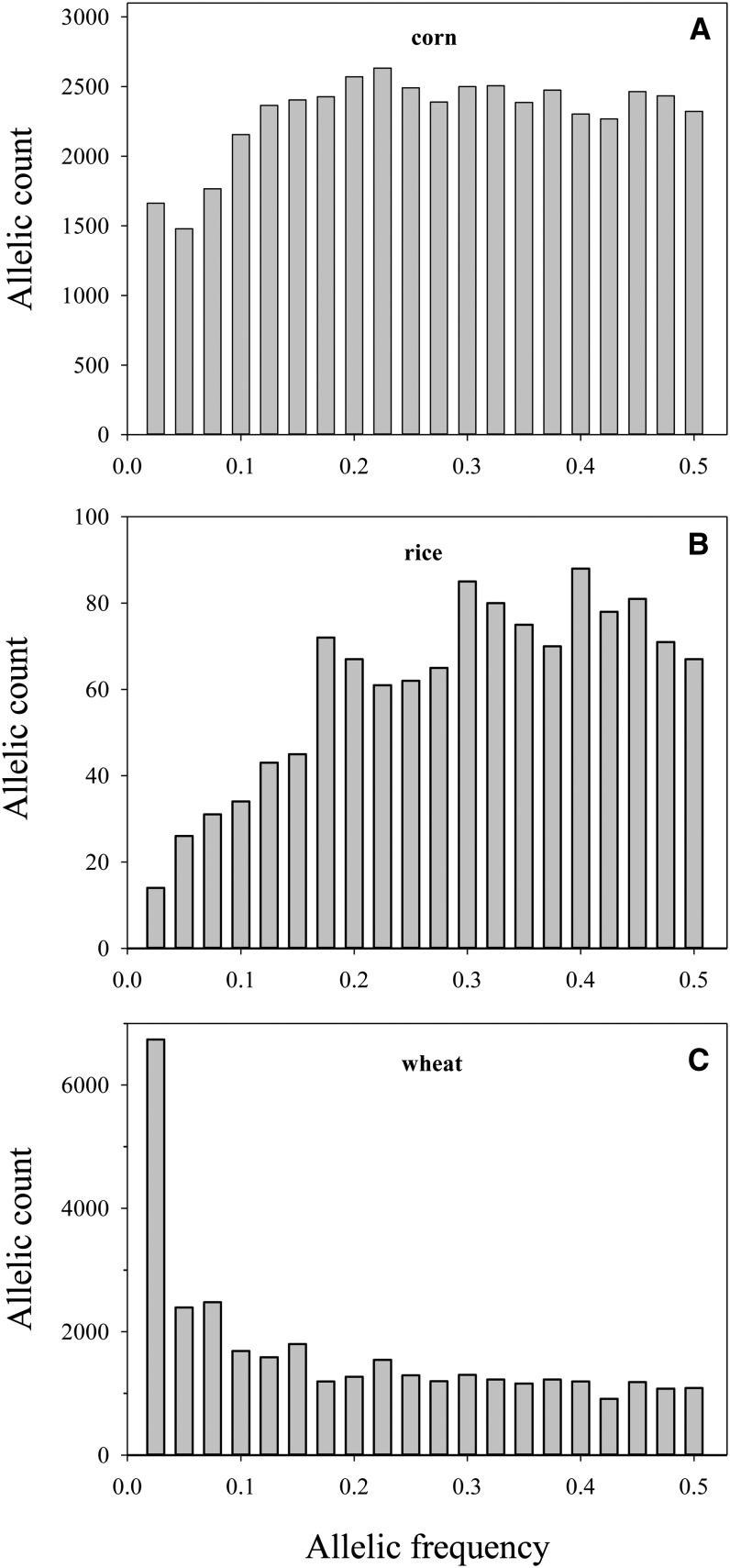
Frequency distribution of the minor alleles in each SNP genotype data set (A: corn data of 400 lines × 45,997 markers, B: rice data of 395 lines × 1311 markers, and C: wheat data of 339 lines × 33,526 markers).

These three original data sets were selected to represent three different crops (corn, rice, and wheat) with different allele frequency distributions (Figure 1). For each crop data set, the minor alleles were determined and their frequencies across the assayed lines were tabulated. The corn data roughly showed a uniform distribution of minor alleles. The rice data revealed a small excess of greater minor allele frequencies with a maximum around 0.45. The wheat data displayed a large excess of lower minor allele frequencies, particularly with less than 0.1. Note that rice alleles were largely genomic SNPs, whereas corn and wheat alleles were associated with transcribed SNPs.

### Diversity estimates under random missing

The diversity estimates for the original data and for those data under random missing ranging from 10 to 90% of its observations without genotype imputations are shown in Table 1. The observed heterozygosity estimates showed little biases in the corn and rice data sets, but slight upward biases with increased standard deviations in the wheat data set. However, the estimates of expected heterozygosity displayed considerable downward biases (up to 33.2% for corn, 39.1% for rice, and 42% for wheat data with the 90% missing level), whereas their standard deviations were largely stable. Similarly, the estimates of inbreeding coefficient also showed some degree of a downward bias (up to 24.1%) for the corn data with the 90% missing level, along with increased variances, but were largely stable for the rice and wheat data. The estimates of genetic differentiations among four groups present in three data sets were less affected, as the Φst statistics were considerably stable over variable levels of missingness, although increased standard deviations were observed at the higher levels of random missing. The estimates of topology accuracy based on the neighbor-joining tree of four representative samples were decreased with increased levels of missingness but were surprisingly robust in the rice and wheat data sets. For example, the topology accuracy remained 80% or greater when 40% of the wheat genotypes was randomly missing and 51% when the missing level increased to 80%. The estimate for the corn data were 50% or greater when the missing level was 30% or lower. However, the estimate for the rice data were relatively low when compared to the other data sets, but remained 50% or greater when the missing level was increased to 80%.

**Table 1:** Estimates (with SD) of observed and expected heterozygosity (Ho, He), inbreeding coefficient (F), Phi statistic (Φst), and topology accuracy obtained from 100 runs of simulation on each SNP genotype data set (corn, rice, and wheat) with 0−90% of total observations as random missing without genotype imputation

Data set/ Missing (%)	Ho (%)*^a^*		He		F		Φst (%)		Topology Accuracy
	Mean	SD	Mean	SD	Mean	SD	Mean	SD	
Corn									
0*^a^*	10.280	0.813	0.334	0.005	0.673	0.024	5.203	0.911	
10	10.283	0.810	0.333	0.005	0.670	0.024	5.182	0.866	57
20	10.296	0.815	0.332	0.005	0.668	0.025	5.182	0.948	62
30	10.326	0.812	0.330	0.005	0.664	0.024	5.313	0.901	54
40	10.249	0.855	0.328	0.005	0.663	0.026	5.112	0.894	47
50	10.284	0.821	0.324	0.005	0.657	0.025	5.081	0.955	45
60	10.234	0.812	0.318	0.005	0.650	0.025	5.312	1.006	44
70	10.302	0.827	0.308	0.005	0.635	0.026	5.231	0.991	43
80	10.351	0.832	0.286	0.005	0.604	0.027	5.157	1.140	37
90	10.211	0.815	0.223	0.005	0.511	0.032	5.052	1.343	21
Rice									
0	0.524	0.085	0.353	0.008	0.983	0.003	4.385	1.922	
10	0.522	0.084	0.352	0.008	0.983	0.003	4.375	1.868	68
20	0.522	0.092	0.350	0.007	0.983	0.003	4.248	1.943	70
30	0.525	0.088	0.348	0.007	0.983	0.003	4.350	1.933	68
40	0.528	0.086	0.344	0.007	0.982	0.003	4.453	1.847	65
50	0.522	0.088	0.339	0.008	0.982	0.003	4.188	1.844	67
60	0.526	0.090	0.333	0.007	0.981	0.003	4.488	1.964	61
70	0.534	0.091	0.320	0.007	0.980	0.004	4.534	1.964	61
80	0.518	0.093	0.291	0.007	0.978	0.004	4.410	1.961	57
90	0.520	0.114	0.215	0.005	0.971	0.006	4.504	2.531	46
Wheat									
0	0.289	0.075	0.200	0.007	0.975	0.008	1.538	0.689	
10	0.289	0.077	0.198	0.007	0.976	0.008	1.546	0.683	91
20	0.287	0.076	0.196	0.007	0.976	0.008	1.501	0.650	88
30	0.288	0.078	0.194	0.008	0.976	0.008	1.597	0.752	82
40	0.285	0.074	0.190	0.007	0.976	0.007	1.575	0.811	80
50	0.292	0.081	0.186	0.008	0.974	0.008	1.607	0.779	73
60	0.291	0.074	0.177	0.009	0.974	0.008	1.478	0.796	63
70	0.293	0.076	0.166	0.008	0.973	0.008	1.521	0.904	65
80	0.300	0.082	0.148	0.007	0.970	0.009	1.514	1.103	51
90	0.309	0.097	0.116	0.005	0.964	0.012	1.484	2.040	42

a The diversity estimates for the original data (*i.e.*, the data with 0% missing level) were obtained from the diversity analyses of the original genotype data corresponding to those 900 missing data sets from random selections of 130 lines with 1000 markers with 10−90% missing observations. No estimates on topology accuracy were made for the original data. The observed heterozygote estimates (Ho) were given in percentage for ease of comparison.

### Diversity estimates from imputed data

**Figure 2:**
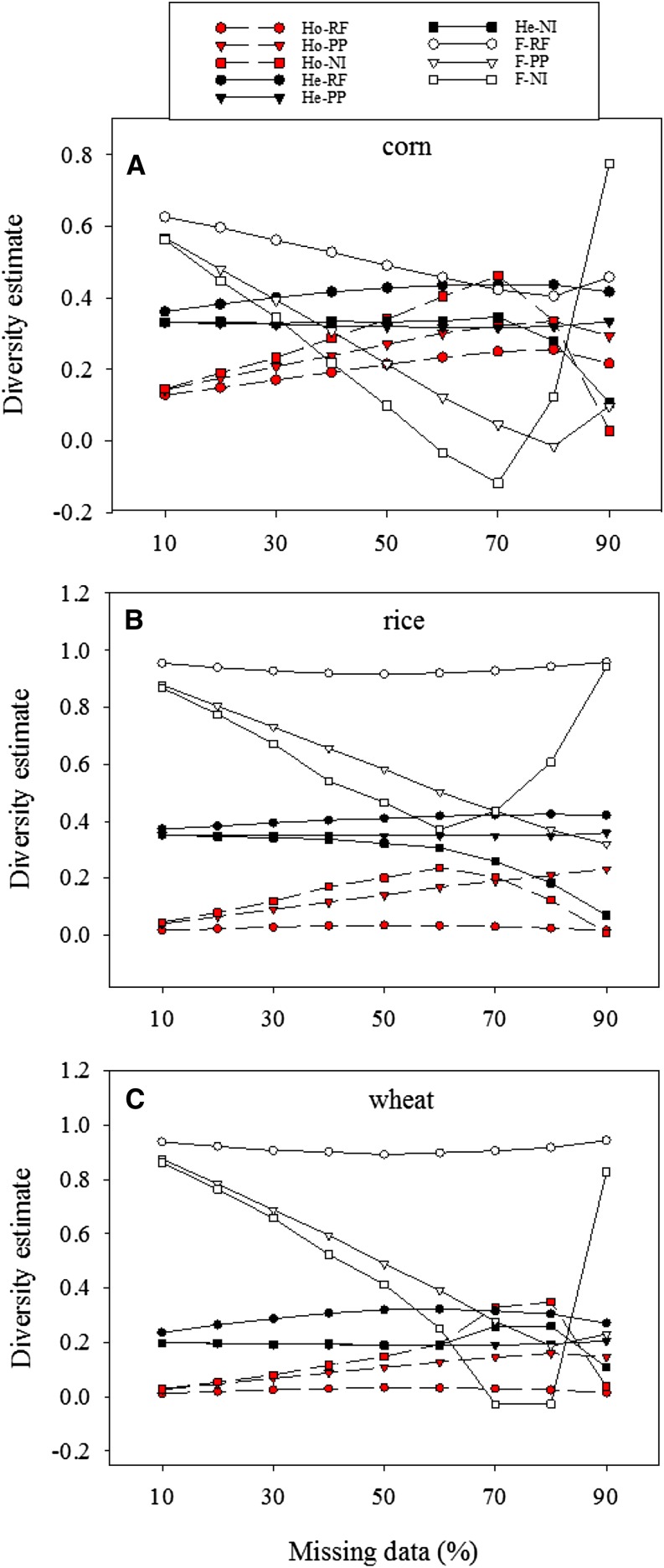
Estimates of observed heterozygosity (Ho), expected heterozygosity (He), inbreeding coefficient (F) over 100 runs of simulation on each single-nucleotide polymorphism genotype data set (A: corn, B: rice, and C: wheat) with 10–90% of total observations as random missing and as imputed with an imputation method. RF, random forest; PP, probabilistic PCA; or NI, nonlinear iterative partial least squares PCA.

The imputation-based estimates of observed heterozygosity, expected heterozygosity, and inbreeding coefficient are shown in Figure 2. These estimates, compared with those without imputations (Table 1), were substantially biased, and the extents of the biases were dependent on the levels of missing data in each crop data set. With increased levels of missing observations, heterozygosity estimates from imputed data were upwardly biased and inbreeding coefficient estimates showed downward biases. The extents of biases for these imputation-based estimations also varied with the crop data sets. When three imputation methods were compared, RF imputation seemed to introduce less bias in diversity estimates than the other two, particularly in the estimates of inbreeding coefficient.

**Figure 3:**
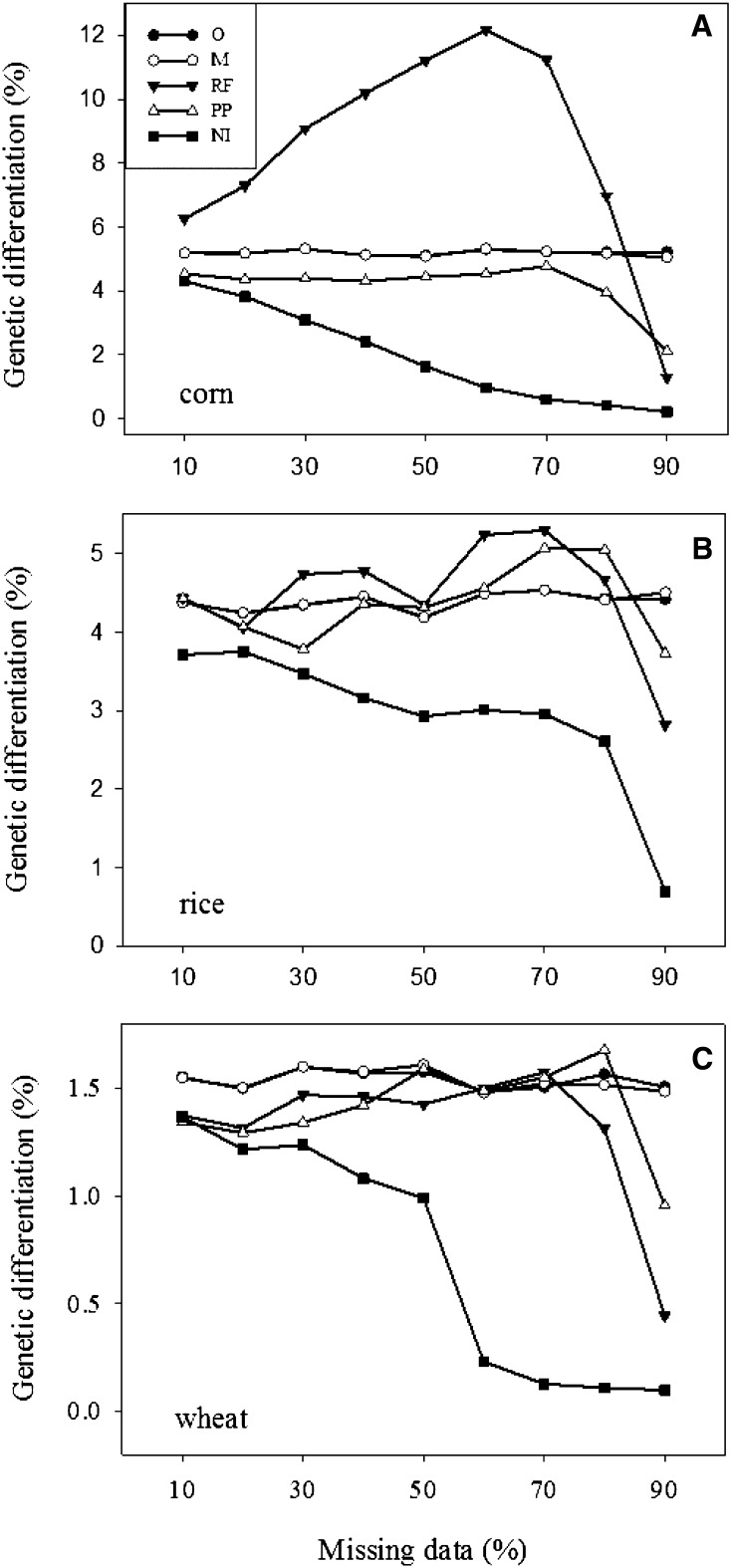
Estimates of genetic differentiations (or Φst statistic) among four groups of 130 individual lines randomly sampled from each SNP genotype data set (A: corn, B: rice, and C: wheat) over 100 runs of simulation with 10–90% of total observations as random missing (M, missing data without imputation) and as imputed with an imputation method (RF, random forest; PP, probabilistic principal component analysis [PCA]; or NI, nonlinear iterative partial least squares PCA). Note that the genetic differentiations also were estimated based on the original SNP data (=O for short) for the corresponding 130 individual lines selected for each simulation run.

The biases in the imputation-based estimates of genetic differentiation, compared with those without imputations (Table 1), also were substantial for the three crop data sets (Figure 3). RF imputation seemed to introduce upward biases, except for the wheat data, whereas NI imputation consistently generated downward biases in all the data sets. However, PP imputation displayed stable levels of bias with increased levels of missingness, except when the missing level was 80% or greater. The extents of the biases observed for the three imputation methods seemed to vary with the assayed data sets.

**Figure 4:**
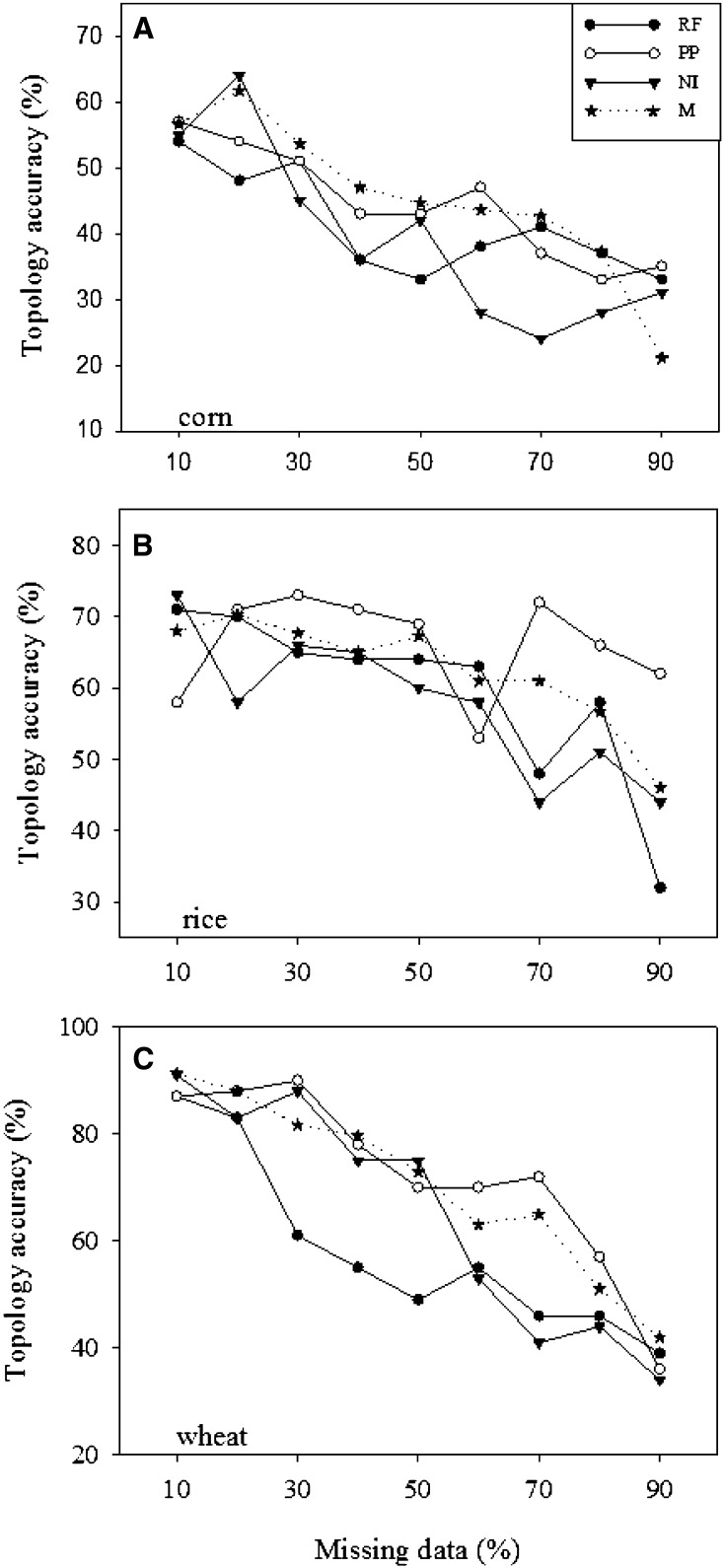
Estimates of topology accuracy (%) for neighbor-joining clusters of four samples representing four groups in each SNP genotype data set (A: corn, B: rice, and C: wheat) over 100 runs of simulation with 10–90% of total observations as random missing (M, missing data without imputation) and as imputed with an imputation method (RF, random forest; PP, probabilistic principal component analysis [PCA]; or NI, nonlinear iterative partial least squares PCA).

The imputation-based estimates of topology accuracy as reflected in four representative samples of interested groups were generally reduced with increased levels of missing genotypes and they were smaller than those from random missing without imputations (Figure 4). However, PP imputation seemed to generate greater estimates of topology accuracy than the other two imputations in the three crop data sets and largely than those without imputations in the wheat data set (Figure 4). The patterns of the topology accuracy estimates also depended on the assayed allelic frequency distributions. For example, the imputations of the wheat genotypes were more effective with relatively greater estimates of topology accuracy than those on the other crop data sets (Figure 4).

**Figure 5:**
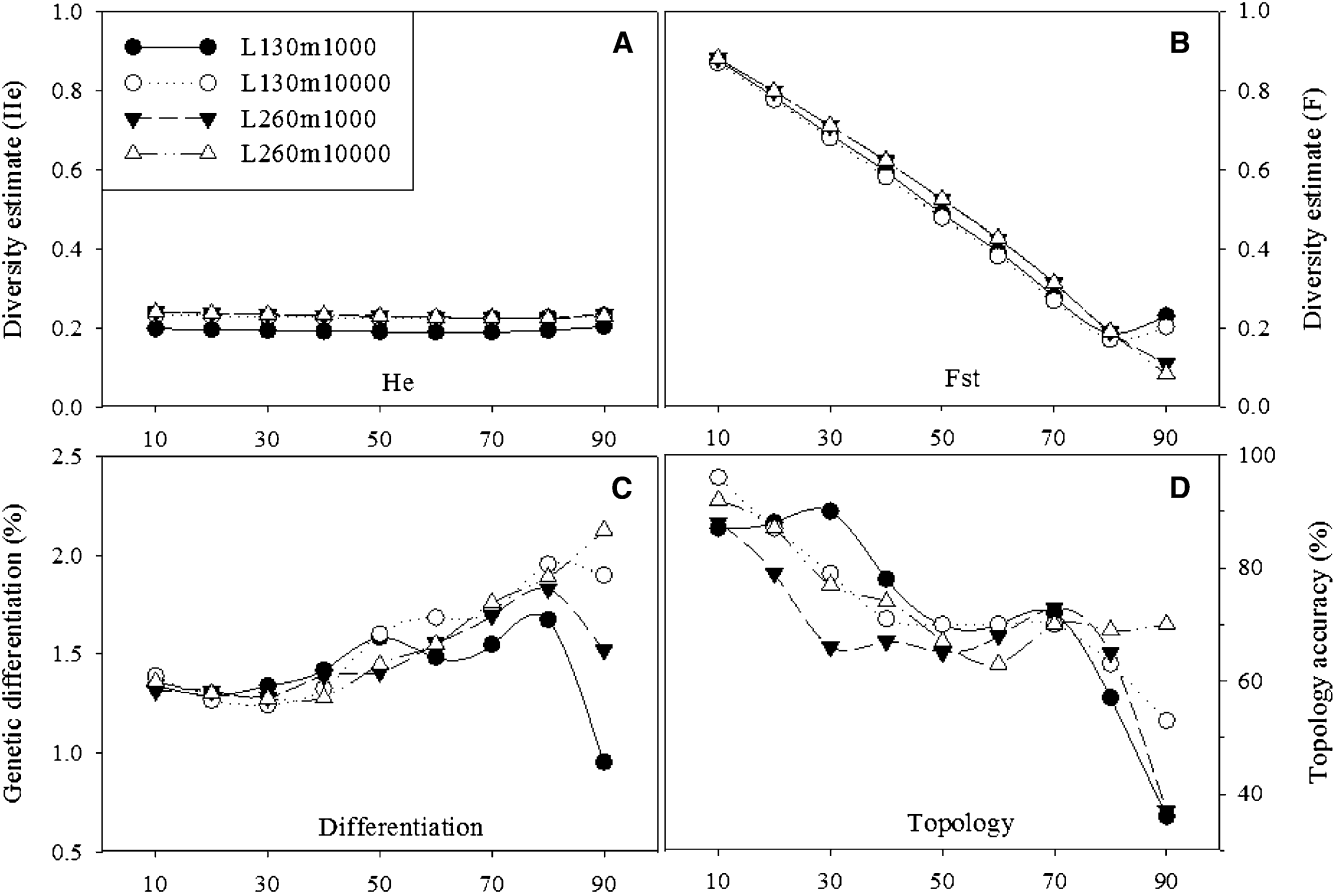
Estimates of expected heterozygosity (He; A), inbreeding coefficient (Fst; B), Phi statistic (Φst; C), and topology accuracy (D) obtained from 100 runs of simulation on the wheat SNP genotype data set with 10–90% of total observations as random missing and as imputed by the probabilistic principal component analysis (PP) imputation for four sample size combinations of breeding lines (L = 130, 260) and SNP markers (m = 1000, 10,000).

Enlarging the sample size from 130 to 260 wheat breeding lines and/or from 1000 to 10,000 markers did not appear to affect much the PP imputation-based estimates of the four diversity parameters (He, F, Φst statistic, and topology accuracy) (Figure 5). Overall, the variations in the diversity estimates among the four sample size combinations were relatively small, except for those in the topology accuracy estimates. The marked differences in the estimates of Φst statistic and topology accuracy were observed when 90% of the wheat genotypes were randomly missing.

### Imputation accuracy and execution time

Another way to assess imputation accuracy is to measure the percentage of the random missing SNP genotypes after imputations in which the imputed genotypes were matched with the corresponding original genotypes. Overall, PP imputation showed the highest estimates of imputation accuracy in the corn and wheat data sets (*i.e.*, 54.4–50.4% and 71.5–67.0% recovery of the original genotypes for the nine levels of missingness, respectively), whereas RF had the highest matches in the rice data set (74.1–65.1% for the nine levels of missingness) (Table 2). More interestingly, PP imputation revealed the greatest recovery of the major (or AA) genotypes, followed by NI and RF imputations. For example, when 10% of the wheat data were randomly missing, PP recovered 67.6% of the original major genotypes, NI 65.5%, and RF 48.4%. In contrast, RF imputation had the greatest recovery of the minor (or aa) genotypes, followed by PP and NI imputations. For example, when 10% of the rice data were randomly missing, RF recovered 23.1% of the original minor genotypes, PP 13.1%, and NI 11.0%.

**Table 2:** Estimates of imputation accuracy as the percentage of the imputed genotypes matched with the corresponding original genotypes over all the random missing SNP genotypes over 100 runs of simulation on each SNP genotype data set (corn, rice, and wheat) with 10−90% of total observations as random missing and as imputed by an imputation method (RF, PP, or NI)

Data Set/Missing (%)	RF				PP				NI			
	M	aa	Aa	AA	M	aa	Aa	AA	M	aa	Aa	AA
Corn												
10	51.3	12.0	7.9	31.3	54.4	2.6	6.2	45.6	50.0	1.6	6.7	41.7
20	50.7	11.9	8.2	30.6	54.2	2.5	6.4	45.2	43.9	1.6	7.8	34.5
30	50.6	11.7	8.0	30.8	54.1	2.5	6.3	45.3	44.7	0.8	7.2	36.6
40	49.8	11.4	8.2	30.1	53.8	2.5	6.4	44.9	42.5	0.4	7.4	34.8
50	48.8	10.9	8.0	29.9	53.8	2.6	6.3	45.0	34.4	0.4	8.3	25.8
60	47.6	10.4	7.7	29.5	53.0	2.6	6.2	44.2	28.7	0.0	8.6	20.1
70	45.8	9.5	6.9	29.4	53.0	2.8	6.0	44.2	14.6	1.1	9.1	4.3
80	43.6	8.2	5.1	30.3	52.2	3.2	5.6	43.3	15.9	10.2	5.6	0.1
90	45.3	7.6	3.0	34.7	50.4	4.6	4.5	41.3	21.1	20.9	0.2	0.0
Asd	1.3	0.5	0.8	1.3	1.3	0.4	0.6	1.2	1.1	0.3	0.7	1.0
Rice												
10	74.1	23.1	0.1	50.9	68.6	13.1	0.3	55.2	62.7	11.0	0.3	51.4
20	73.8	23.0	0.1	50.7	68.5	13.1	0.3	55.2	56.3	10.8	0.4	45.1
30	73.8	23.0	0.1	50.7	68.3	13.0	0.3	55.0	51.2	7.0	0.4	43.9
40	73.6	22.9	0.1	50.5	68.1	13.1	0.3	54.8	38.5	7.1	0.4	30.9
50	73.8	22.9	0.1	50.9	68.3	13.2	0.3	54.9	29.0	3.5	0.5	25.1
60	73.8	22.7	0.1	51.0	66.9	12.6	0.3	54.0	13.8	5.0	0.5	8.3
70	73.7	22.7	0.1	51.0	67.7	13.2	0.2	54.2	5.3	1.9	0.5	2.9
80	72.4	21.9	0.0	50.5	66.5	13.1	0.2	53.2	15.2	14.9	0.2	0.1
90	65.1	18.2	0.0	46.9	61.2	12.7	0.2	48.3	28.4	28.3	0.0	0.0
Asd	1.4	0.6	0.0	1.0	1.6	0.8	0.1	1.0	0.8	0.5	0.1	0.6
Wheat												
10	61.4	12.8	0.1	48.4	71.5	3.7	0.2	67.6	68.6	2.8	0.3	65.5
20	61.4	12.6	0.1	48.6	71.7	3.7	0.2	67.8	64.3	2.9	0.3	61.1
30	60.9	12.4	0.1	48.4	71.5	3.7	0.2	67.6	63.8	1.7	0.3	61.8
40	60.8	12.1	0.1	48.7	71.2	3.5	0.2	67.4	58.4	1.2	0.3	56.9
50	61.6	12.1	0.1	49.5	71.1	3.7	0.2	67.2	52.7	0.7	0.3	51.6
60	62.8	11.8	0.1	50.9	70.5	3.8	0.2	66.5	44.6	0.1	0.4	44.2
70	63.9	11.3	0.1	52.5	70.7	4.0	0.2	66.5	15.7	0.8	0.4	14.4
80	66.0	10.6	0.0	55.4	69.8	4.3	0.2	65.3	8.3	7.7	0.3	0.4
90	67.0	8.1	0.0	58.9	67.0	4.6	0.1	62.2	17.2	17.2	0.0	0.0
Asd	1.4	0.6	0.1	1.4	1.7	0.4	0.1	1.6	1.5	0.3	0.1	1.4

RF, random forest; PP, probabilistic principal component analysis, NI, nonlinear iterative partial least squares PCA); M, the percentage of the imputed genotypes matched with the corresponding original genotypes over all the random missing SNP genotypes; aa, Aa, or AA, the percentage of the specific imputed genotypes matched with the corresponding original minor (aa), heterozygous (Aa), or major (AA) genotypes over all the random missing SNP genotypes, respectively; Asd, the average of the SDs obtained for an accuracy estimate over the nine levels of missingness.

The times for execution of R scripts in the Linux server to assess topology accuracy in the corn data with 100 runs of simulation were recorded as 70.5 hr for RF imputation, 7.08 hr for PP imputation, and 1.1 hr for NI imputation. Some variation in execution time was observed for each imputation method with respect to other diversity estimation and crop data set, but the above trend of variation in execution time remained the same.

## Discussion

The empirical assessment performed here revealed variable patterns of bias in estimation of crop genetic diversity with respect to large missing data and map-independent imputation. Generally, a genetic diversity analysis of a random missing data yielded much smaller biases than that of an imputed data. The estimation of genetic differentiation via Φst statistic was robust up to 90% missing observations but could yield a substantial bias from genotype imputation. The imputation-based estimates of topology accuracy for four representative samples were generally reduced with increased levels of missing genotypes. The probabilistic PCA-based imputation could perform better in topology accuracy than those analyses of missing data without imputation. These findings are significant for understanding the reliability of a genetic diversity analysis with respect to large missing data and map-independent imputation.

The robust estimations of genetic differentiation from nonimputed data with respect to high incompleteness are expected because the algorithm used to calculate Φst statistic takes into account missing observations (Excoffier and Lischer 2010). For some applications, alleles of frequency less than 0.05 may be excluded, but our analysis included those genotypes of frequencies less than 0.05, as we followed the co-dominant genotype data to estimate Φst statistic. However, our nonimputation-based estimations of heterozygosity and inbreeding coefficient also considered the missing observations but still yielded considerable biases (Table 1), although much smaller than those from genotype imputation (Figure 2). The reason for this remains unknown. It is possible that the biases were due to either nonrandomness partition by imputation of diversity among four groups or other errors we did not detect from genotype imputation. Our assessment showed that PP imputation outperformed the other two methods both in the recovery of missing genotypes and the inference of genetic relationships. This was somehow surprising, given the favorable assessment done on RF for genomic selection (Rutkoski *et al.* 2013). This could be explained with the specific finding that PP imputation was mostly effective in recovering the major genotypes, whereas RF was the best in recovering the minor genotypes (Table 2). However, with respect to imputation speed, NI imputation was the fastest, while RF imputation was extremely slow.

This assessment revealed two interesting findings. First, the assessed imputations did not show any superiority in accuracy for these genetic diversity analyses (Figure 2, Figure 3, and Figure 4) over those without imputations (Table 1). The traditional genetic diversity analyses of highly incomplete genotype data showed less biased in diversity estimates than those analyses of imputed data. Second, the high PP imputation accuracy helped only the genetic relationship inference (Figure 4), but not much the estimations of heterozygosity, inbreeding coefficient and genetic differentiation (Figure 2 and Figure 3). For example, PP imputation recovered 67% of the original genotypes for up to 90% level of missing data (Table 2), but substantial biases were still observed in the imputation-based estimations of heterozygosity, inbreeding coefficient and genetic differentiation (Figure 2 and Figure 3). It is possible that the biases were largely due to the loss of the minor genotypes from random missing, as PP imputation was mostly effective in recovering major, not minor, genotypes (Table 2).

However, our assessment had several limitations. First, our findings may be data specific, as the assessed types of allele frequency distribution may not reflect those present in GBS data and for other organisms. These crop data sets may differ from GBS data in the generation of SNP data and the sampling coverage of a genome, as mentioned above. Deviations from GBS allele frequency distributions may generate patterns of bias different from our findings. Second, although we have examined a wide range of missing data from 10 to 90% compatible with the reported GBS data (Elshire *et al.* 2011; Fu and Peterson 2011; Fu *et al.* 2014), our analyses were limited to those assayed imputation methods, essentially only two types of imputation using correlation information. More assessments could be explored with other imputation methods and different types of allele frequency distribution to enlarge the scope of accuracy assessments with respect to high incompleteness. Third, it is difficult to assess topology accuracy for a large number of samples (see Robinson and Foulds 1981) and we proceeded with only four samples as Wiens and Tiu (2012) did for a phylogenetic analysis. These topology accuracy measures provided only a snap-shot, but not necessarily the whole picture, of accuracy in the genetic relationship inferences of 130 samples. Fourth, our assessment considered only the traditional methods commonly used in genetic diversity analysis, not those maximum likelihood or Bayesian methods recently developed for population genetic inferences from NGS data (see Nielsen *et al.* 2011). The latter may help to reduce estimation bias due to low coverage like those reported in GBS data (*e.g.*, see Fumagalli *et al.* 2013). Fifth, we considered only one simple missing mechanism: random missing. The real mechanisms for missing observations from GBS may be complicated and the expected patterns of estimation bias could differ from what were reported here. Simulating GBS data for different organisms may help to shed more light on the impact of highly incomplete genotype data on diversity estimation.

Despite these limitations, the findings reported here have some implications for the genetic diversity analysis of large unbalanced SNP genotype data, particularly for those highly incomplete GBS data acquired from nonmodel species. First, the estimation of genetic differentiation via Φst statistic was robust, requiring no imputation for missing genotypes. Second, the estimations of heterozygosity and inbreeding coefficient would become less accurate with larger numbers of missing observations, but the estimation biases were much smaller than those from genotype imputations. Thus, these diversity estimations from genotype imputations should not be encouraged. Third, the probabilistic PCA-based imputation could enhance topology accuracy in genetic relationship inferences, but may not always yield higher topology accuracy than those from missing data without genotype imputation. Fourth, considering those assayed types of allele frequency distribution (Figure 1), one could reason that these findings may also be applicable to some highly incomplete GBS genotype data generated from non-crop organisms. Last, the demonstrated estimation biases imply more efforts are needed to (1) minimize genotype incompleteness with more effective GBS protocols (Sonah *et al.* 2013); (2) develop more effective map-independent imputation tools for GBS data (Poland *et al.* 2012); (3) integrating a GBS protocol with *de novo* sequencing to assemble a reference for generating reference-based GBS data for which map-dependent imputation can be applied; or (4) develop more accurate population genetic inferences from highly incomplete GBS data (Lynch 2008; Nielsen *et al.* 2012; Fumagalli *et al.* 2013).

In conclusion, these findings indicate that the genetic diversity analysis of highly incomplete GBS genotype data could be reliably performed but requires caution with respect to analysis goal, missing level and imputation application. The findings reported here are also instructive for performing a proper genetic diversity analysis of highly incomplete GBS or other genotype data.

## Supplementary Material

Supporting Information
