# Supplementary material for: Genetic Diversity Analysis of Highly Incomplete SNP Genotype Data with Imputations: An Empirical Assessment
Source: G3 (Bethesda). 2014 Mar 13;4(5):891–900. doi: 10.1534/g3.114.010942 (PMC4025488; doi:10.1534/g3.114.010942)
Supplement: Supporting Information [file supp_4_5_891__index.html]

Genetic Diversity Analysis of Highly Incomplete SNP Genotype Data with Imputations: An Empirical Assessment — Supporting Information 

# Genetic Diversity Analysis of Highly Incomplete SNP Genotype Data with Imputations: An Empirical Assessment

## Supporting Information for Fu, 2014

**Files in this Data Supplement:**

- File S1 - Corn dataset (.csv, 35 MB)
- File S2 - Rice dataset (.csv, 1 MB)
- File S3 - Wheat dataset (.csv, 22 MB)
